# Combining CNN and Q-learning for increasing the accuracy of lost gamma source finding

**DOI:** 10.1038/s41598-022-06326-0

**Published:** 2022-02-16

**Authors:** Atefeh Fathi, S. Farhad Masoudi

**Affiliations:** Department of Physics, K.N. Toosi University of Technology, P.O. Box 15875−4416, Tehran, 15418−49611 Iran

**Keywords:** Engineering, Physics

## Abstract

The increasing use of nuclear technology in various fields makes it necessary to provide the required safety to work with this industry. Gamma source is one of the most widely used sources in industry and medicine. Finding a lost gamma source in a gamma irradiation room without human presence is challenging due to the particular arrangements and barriers in the room for radiation shielding and requires an efficient and robust method. In this paper, locating and routing the lost gamma source in the gamma irradiation room containing radiation blocking barriers are done simultaneously by using two methods, convolutional neural network (CNN) and Q-learning, which are powerful algorithms for deep learning and machine learning. Environment simulation with gamma source was performed using Geant4 simulation. The results show that by combining these two methods in geometries with radiation blocking barriers, in addition to locating with 90% accuracy, routing can also be performed. Although the presence of thick barriers in the room reduces the accuracy, increases the time required to finding the lost gamma source or the inefficiency of other methods, nevertheless, the results show that combination of CNN and Q-learning reduces the time and greatly increases the accuracy.

## Introduction

Gamma radiation is widely used in industry and medicine. However, high radiation exposure can cause definite and accidental effects on the human body. If a radiation source is lost, it can be potentially harmful to people, especially those looking for it. So, robots are used to detect the lost radiation source to reduce the potential risk to humans and the environment. Robots play a significant role in radiation environments that are inaccessible to humans. With the development of the nuclear industry and the technology of robots, the performance of the radiation detection robots expanded. Different detector design techniques and systems have been used to monitor the radiation by robot and detect radioactive sources^1–5^. For example, a robot with dimensions of 150 cm × 150 cm × 80 cm and a weight of 600 kg was used along with a new detector design for radiation intensity detection and 3D imaging, humidity, and temperature detection^6^. Lin et al. used the artificial potential field approach to search the environment to assess the location and amount of radiation from a lost source without having an environmental map^7^. Hjerpe presented a statistical method for locating a lost radiation source by studied different detector geometries and investigated the radioactive source^8^. Wilhelm et al. used Monte Carlo-based software, MCNP, to explore the environment in conjunction with the NASVD method^9^. Sharma et al. developed an algorithm for locating a ^137^Cs source in a three-dimensional space using various detector measurements at different locations^10^. Many methods have been reported for estimating the radiation source location using a single radiation detector^11–14^. However, an additional detector is needed to find the exact location of the radiation source^15–20^. The detector system used in these studies is expensive and usually very heavy, making it impossible to fit in small robots. A new algorithm is needed to use only a simple and inexpensive detector that can search the location of radioactive sources quickly and accurately. Many research groups have designed and built a detection system with one or more detectors to find the lost gamma source^18,20–23^. Designing and building a new detection system requires extra facilities and, of course, economic costs. Here, the detector system is simple and accessible so that it can be moved easily, like a regular NaI(Tl) detector, but a robust location algorithm is used.

Today, machine learning is used in many different sciences. Machine learning is a subset of artificial intelligence that automatically allows systems to learn and progress without explicit programming^24^. Deep learning and reinforcement learning are two other subsets of machine learning. Deep learning is the same as machine learning in such a way that, for complex examples, it does the learning for the machine, and in this way the machine gains a better understanding of existential realities and can identify different patterns^25^.

Deep learning has achieved a very high level of accuracy in diagnosis. Recent advances in deep learning make it works better than humans in some respects. For example, recent work has been shown that by using deep learning, the location of the lost gamma source can be identified with high accuracy, only by measuring the energy deposition at five random and sequential points. It investigated the convolutional neural network (CNN) algorithm to find a lost gamma source in a simple room and a room with one and two walls^26^. Although the lost gamma source can be found by the proposed algorithm, however, the wall thickness has been considered 50 cm, which attenuates the radiation behind the wall and does not block it. The results of this research showed that the thicker the wall inside the room, the less accurate the algorithm.

In nuclear installations, in rooms where nuclear radiation is used, thicker shields and walls are used so that the nuclear radiation behind them is completely blocked^27^. Therefore, finding a lost source in such rooms will be challenging because the pattern of source radiation distribution will be unpredictable due to the presence of thick walls and the CNN algorithm alone cannot be very accurate in such patterns. Therefore, one should look for an algorithm that can recognize the environment based on experience and make decisions based on the response it receives from the environment.

In such cases, we proposed using Reinforcement learning in which, an agent learns in an interactive environment, by using trial and error and feedback from previous actions and experiences. An agent takes decisions based on earned knowledge. Here, the agent is considered a set of a robot, a detector, and an information processing system. This method is appropriate for all issues that decision sequences are essential, such as routing in the room with a thick maze. Q-learning is a reinforcement learning technique that follows a specific policy for performing different movements in different situations by learning an action/value function. One of the advantages of this method is learning the function without having a specific environment model.

**Figure 1:**
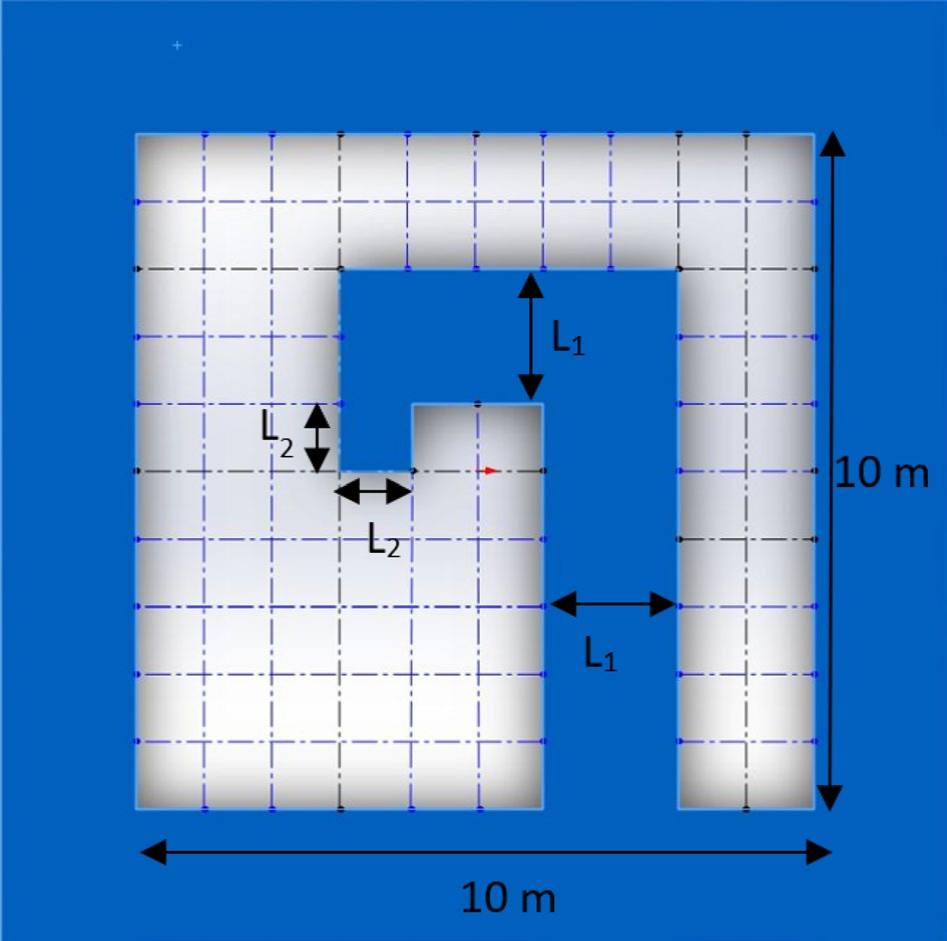
The Geometry of the room. The room is divided to 77 cells with 100 × 100 cm dimensions. The center of each cell is considered for the position of the gamma source. For studying the effect of wall thickness, three cases are considered for L_1_ and L_2_: (L_1_ = 50 cm, L2 = 50 cm), (L_1_ = 100 cm, L_2_ = 100 cm) and (L_1_ = 200 cm, L_2_ = 100 cm).

The purpose of this research is to find a lost gamma source in a real room geometry at a nuclear facility. The geometry used in this article is similar to the gamma irradiation room geometry in Tunisia. The approximate dimensions of the room are derived from^27^ and shown in Fig. 1. The scenario is as follows: a gamma source with 2 MeV energy and activity of 2 µCi is lost in this room. An agent automatically starts measuring radiation at points with 50 cm distances from each other and uses them as input data of the proposed algorithm.

CNN and Q-learning methods have been used to find and routing the gamma source simultaneously. In principle, the location of the gamma source is estimated in several steps by the CNN method, and for routing, the Q-learning method is used. During the movement, in several steps, the location of the gamma source is re-estimated with new measurements to increase the accuracy. If the prediction of the network changes, the route will be performed according to the new prediction. In the following, first, the method of predicting the location of the source using CNN and then the routing method using Q-learning are explained. Also, the combination of these two methods is mentioned.

## GEANT4 simulation

The first step of the CNN method is the generation of a data set, which will be the input for CNN. Here, GEANT4 simulations are used for generation of the data set. According to Fig. 1, the floor of the room is divided into 77 cells with 100 × 100 cm dimensions. By considering the gamma source in the center of each cell, the radiation energy deposition map has been simulated using the GEANT4 program using 40 × 40 matrix. The pixels dimension of these matrices is 25 cm × 25 cm.

**Figure 2:**
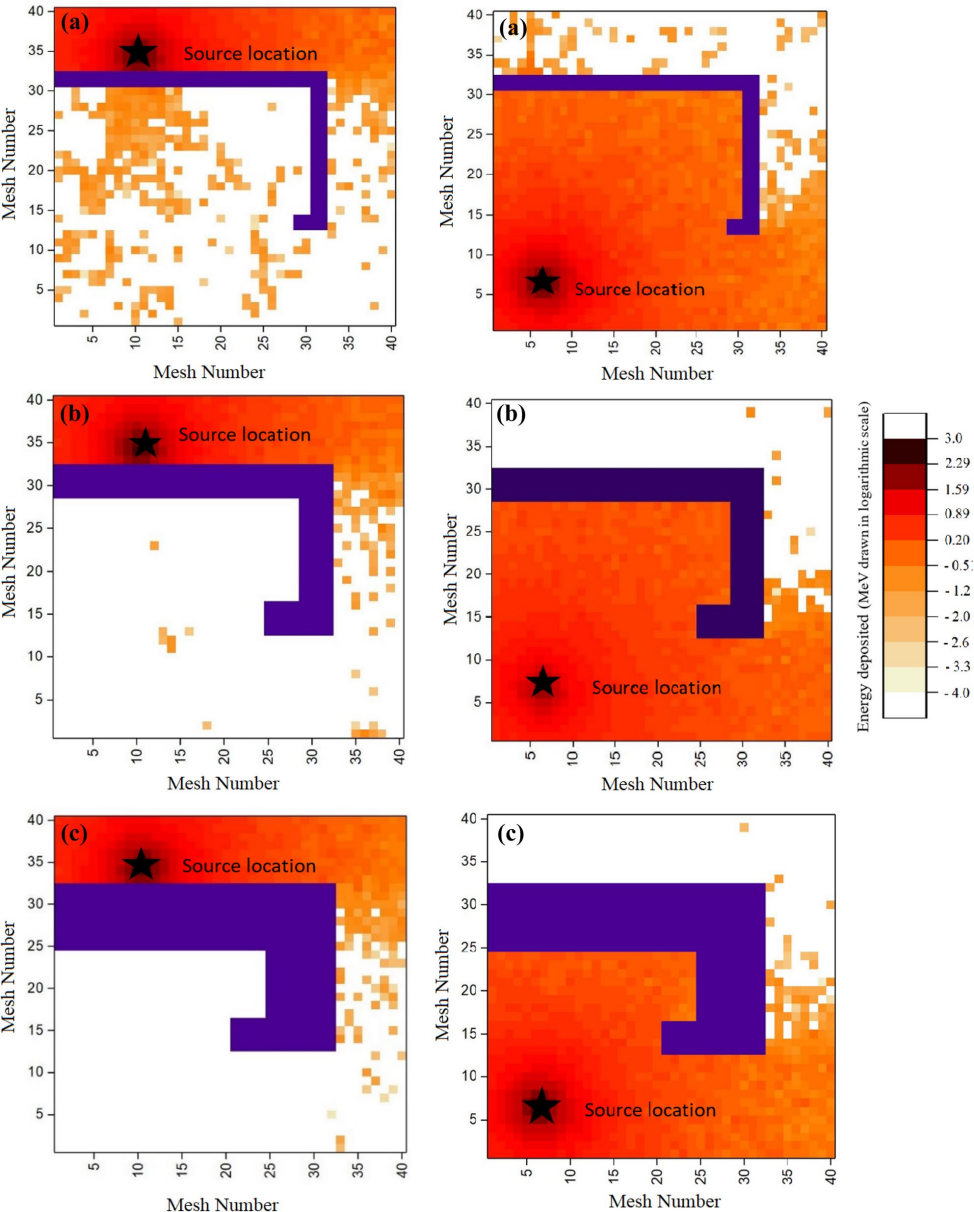
Gamma source energy deposition map (MeV drawn in logarithmic scale) calculated by Geant4 simulation located in a meshed room (dimension of each mesh is 25 cm × 25 cm) with a walled maze with thickness: (**a**) (L1 = 50 cm, L2 = 50 cm), (**b**) (L1 = 100 cm, L2 = 100 cm) and (**c) **(L1 = 200 cm, L2 = 100 cm).

The room has a thick wall that ensures that the radiation does not reach the back of the wall during treatment. However, as the thick wall in the middle of the room blocks much of the radiation, it makes challenging to locate the lost gamma source in this geometry. Figure 2, shows several radiation maps obtained from the Geant4 simulation considering different gamma source locations inside the room with a walled maze with thicknesses 50 cm, 100 cm and 200 cm.

## CNN method

A convolutional neural network (CNN) is a deep learning algorithm that receives an image input.

A CNN uses convolutional mathematical operations instead of general multiplication of matrices in at least one of its layers^28^. The purpose of CNN is also to use the information between the pixels of the image. Due to the characteristics and power of the convolution neural network, this neural network can be used to predict the location of a lost gamma source with high accuracy. In the following, the steps of learning CNN are explained. Additional information is given in Ref.^26^.

After simulation by the Geant4 program, 77 matrices will be obtained, representing the energy deposition map in the room for 77 different gamma source locations. The agent is assumed to move only in the four main directions up, down, left, and right, and the distance between the consecutive measuring points is 50 cm.

Paths with different lengths are obtained from each of these radiation maps, including measuring the deposited energies in:

- 5 points at distances of 50 cm from each other along a path of 200 cm long.
- 10 points at distances of 50 cm from each other along a path of 450 cm long
- 15 points at distances of 50 cm from each other along a path of 700 cm long
- 20 points at distances of 50 cm from each other along a path of 950 cm long
- 25 points at distances of 50 cm from each other along a path of 1200 cm long.

Many of these paths are made completely randomly from each of the matrices of the radiation energy map. According to the results of Ref.^26^, using 3000 data for each classification group has a better result. Since 77 classes are defined in this article, 231,000 matrixes as datasets have been used for each path length, i.e., from each of 77 radiation maps, 3000 random paths with specified length have been made. Input data was divided into two parts; 20% of the input data were selected as test data, and 80% were considered as training data. The training data was divided into two parts; 30% for network evaluation during training and 70% for network training.

**Figure 3:**
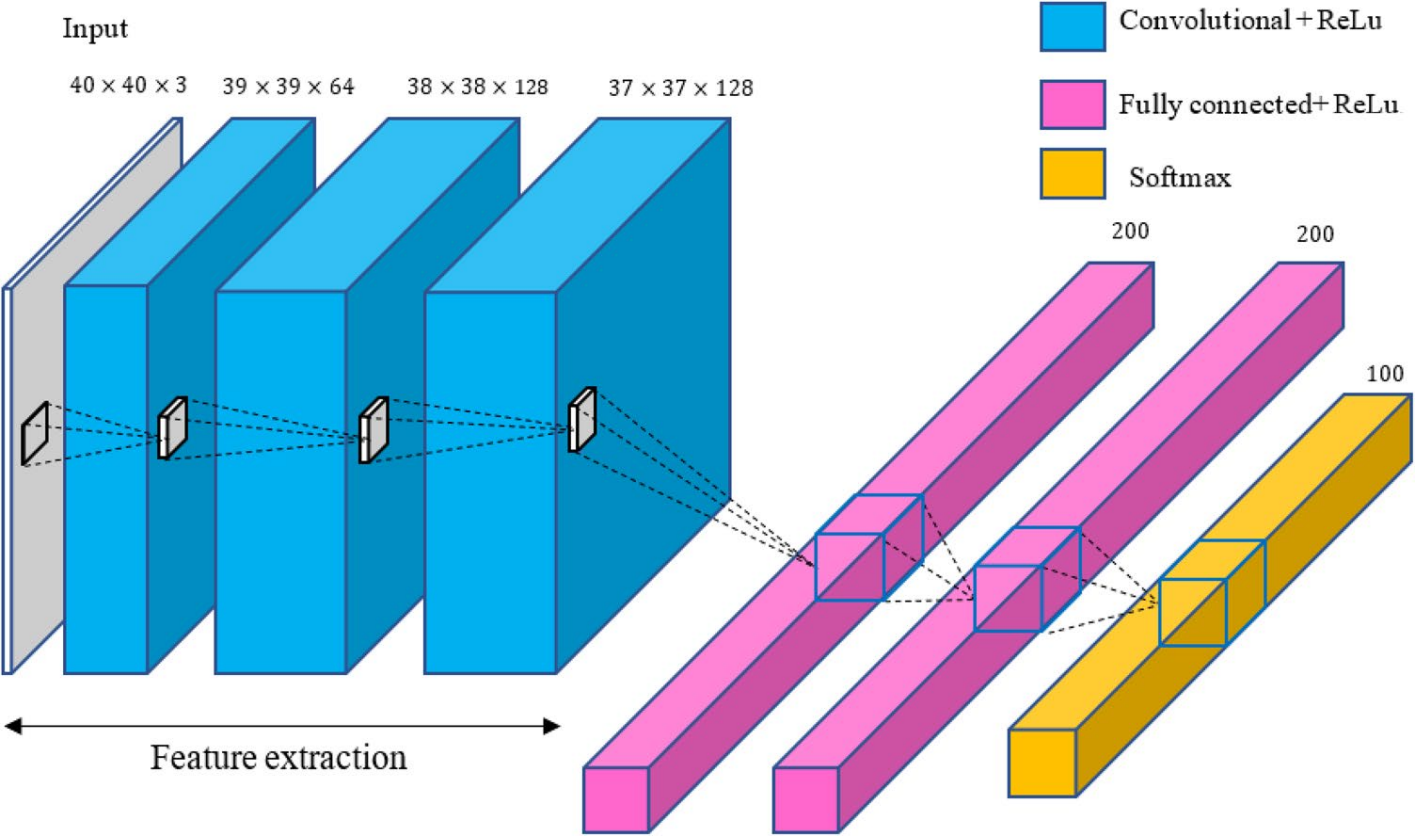
The convolutional neural network architecture in the proposed method.

Figure 3, shows the CNN structure used in the proposed method.

The CNN structure includes an input layer, three convolutional layers, non-linear activation function and three fully connected layers. Matrixes enter the CNN with this structure and the operations are performed in each of these layers. In the end, a class or percentage of occurrence of several different classes is displayed on the output. More information is available in^26^.

The CNN performance on the test dataset should be evaluated during and after training. Different metrics should be used to evaluate CNN. Accuracy, Loss, precision, F1 scores and recall are standard metrics. The brief definitions of these parameters are explained in the following. The ratio of the number of correct predictions to the total number of input samples is Accuracy which is used to measure the neural network performance. The difference between the actual value and the predicted value of the network is the loss function. In the training process of the neural networks, the network's parameters are optimized in each epoch for minimizing the loss. The ideal value of the loss is close to zero. An epoch is one pass over the entire dataset in the neural network.

The fraction of correct detection by the neural network is precision. The fraction of the true detected events by the neural network is recall, and F1 score is an average of Precision and Recall, which gives equal weight to Precision and Recall.

The CNN with the mentioned structure is trained for different datasets that are built for different gamma locations for three rooms with a walled maze with 50 cm, 100 cm and 200 cm thickness. The results of the CNN evaluation are given in the Table 1.

**Table 1:** The evaluation results of the CNN model for room with walled maze with 50 cm, 100 cm and 200 cm thickness.

Wall thickness	Step	Precision	Recall	F1-score	Accuracy
50 cm	5	0.72	0.72	0.73	0.74
	10	0.81	0.82	0.81	0.81
	15	0.86	0.85	0.86	0.85
	20	0.88	0.87	0.87	0.88
	25	0.90	0.89	0.89	0.89
100 cm	5	0.50	0.51	0.51	0.52
	10	0.56	0.56	0.56	0.56
	15	0.58	0.57	0.57	0.58
	20	0.64	0.63	0.64	0.63
	25	0.66	0.67	0.66	0.66
200 cm	5	0.47	0.48	0.47	0.48
	10	0.55	0.54	0.54	0.54
	15	0.58	0.58	0.58	0.59
	20	0.62	0.62	0.61	0.62
	25	0.64	0.65	0.65	0.65

The results of Table 1, show that CNN is a suitable method to find the lost gamma source in a room with a walled maze 50 cm thick. The CNN could predict the lost gamma source location with 74 percent accuracy by a 5-step path and 88 percent accuracy by a 20-step path. However, for thicker walls, such as 200 cm, the CNN accuracy is 48 percent for a 5-step path and 62 percent for a 20-step path. In other words, increasing the wall thickness from 50 cm to 2 m reduces the accuracy by more than 25%.

These results show that in the geometry with thick walls, the CNN is not enough alone to determine the location of the lost gamma source in a short time and with high reliability. We proposed to use the Q-learning method for increasing the accuracy of the lost gamma source finding.

## Q-learning method

Reinforcement learning is a type of machine learning in which an agent, through interaction with the environment, tries to perform a behavior, or in other words, learns how to deal with the environment. Reinforcement learning is based on trial and error. This method is used for all issues in which the decision sequence is essential. The agents learn how to move from the start state to the state that is closer to the goal. The aim is to find the shortest path and the best path to reach the goal from the beginning of the movement.

Reinforcement learning is a machine learning technique in which an agent learns to perform in the environment by interacting with it and getting feedback from its actions and experiences. In Q-learning, each agent has a state in the current time and takes action based on its policy function. State and action are considered as a regular pair of < operations, states > and the value of Q (x_i_, a_i_) is attributed to them^29^. This value is maximum expected reward the agent received when moves from x_i_ state with a_i_ action to the next modes following the policy. How an agent acts at any given time is called that agent's policy. According to the intended purpose, policy determines which of the actions is better to be performed at any time. Q-learning algorithms use tables or matrices, often with initial values of zero. Only one target is selected in the environment, and when the agent reaches the target or fails, one episode ends, and another begins with a random initial state. The Q value is updated by the following equation^29^.

1 $$Q^{new} \left( {x_{i} ,a_{i} } \right) = \left( {1 - \alpha } \right)Q^{old} \left( {x_{i} ,a_{i} } \right) + \alpha \left( {{\text{r}}_{i} + \gamma \mathop {\max }\limits_{u} Q\left( {x_{i} ,a_{i} } \right)} \right)$$

Equation 1 is known as the Bellman Equation which is the core of the Q-learning algorithm. In which, the learning rate α ∈ (0,1) determines the preference of the obtained information over the previous information. Thus, a zero-learning rate means that the agent does not learn anything new, and a learning rate equal to one means that the agent uses only new information as a criterion^30^.

The discount factor γ ∈ [0,1) in Eq. (1), determines the importance of future rewards. The zero value of γ causes the agent to be greedy or short-term and only considers current rewards and values close to one, encourages the agent to struggle for a longer period for the reward^30^. If the discount factor is one or more than one, the values diverge^31^. From zero knowledge of the environment, the agent tries an action a, observes what happens in form of reward, r, and next state. $\mathop {\max }\limits_{u} Q\left( {x_{i} ,a_{i} } \right)$ chooses the next logical action that will give the maximum Q value for the next state. The Q value for that current state-action pair is updated. Doing the update iteratively will eventually learn the Q value function^32^.

In our method, the agent must find the best route to reach the gamma source. The agent can move as much as two meshes at any time (50 cm). The goal is for the agent to reach the gamma source with less radiation measurement number and thus spend less time. The strategy used to reach the gamma source is to use the Q-table. In the Q-table, the maximum expected future reward for an agent is calculated in each state. The best possible action for each state is determined by the Q-table. Finding a way to get closer to the gamma source helps the agent choose a better path to measure the deposited gamma energy, so the CNN estimates the location of the gamma source more accurately.

**Figure 4:**
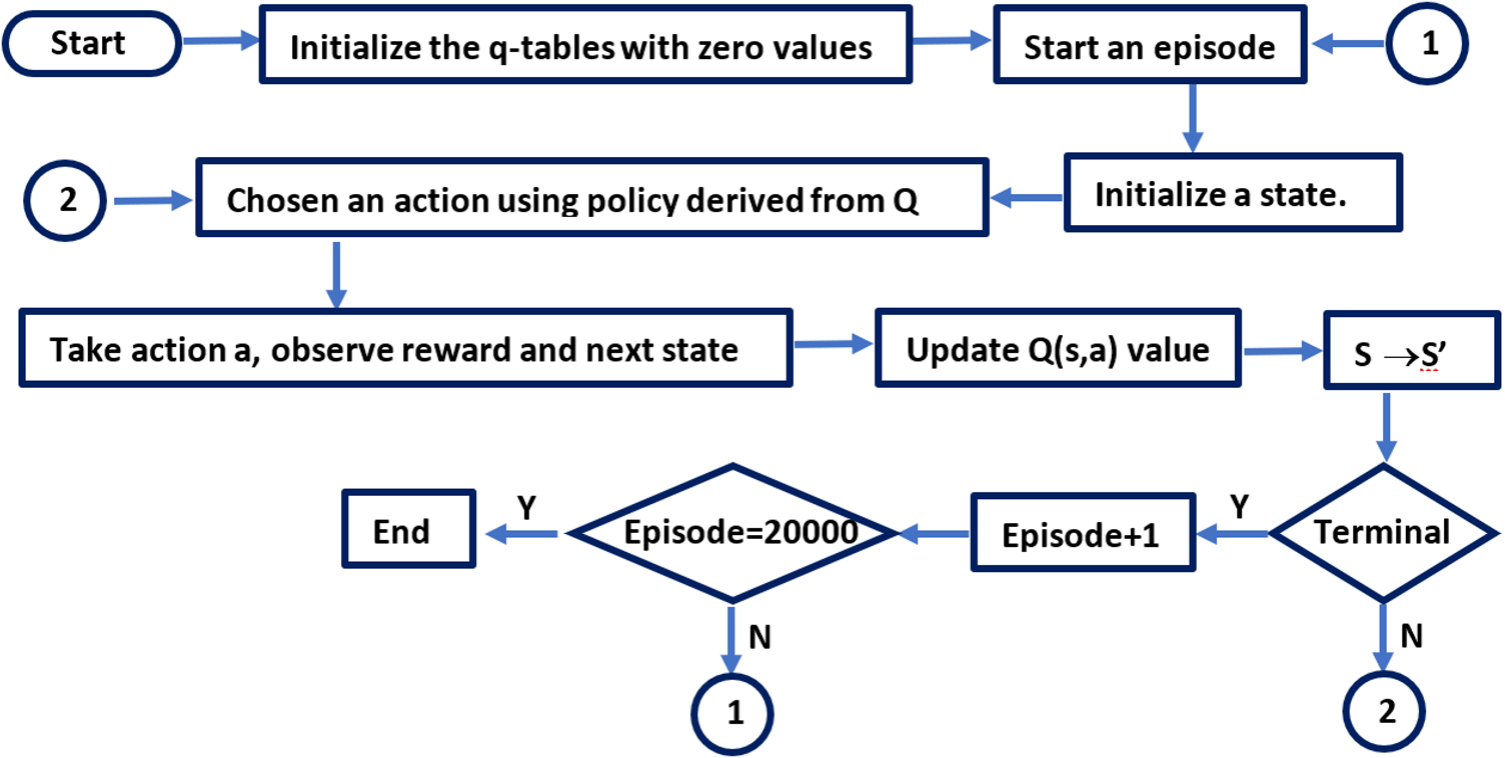
Diagram of Q-learning in the proposed method.

As previously mentioned, the room is divided into 1600 meshes. Meshes are considered as states, which an agent can move in four directions (up, down, left, and right) in each state. The rows of the Q-table are equal to the number of states, and its columns are equal to four actions. Therefore, the Q-table has 1600 rows and four columns. The value of each Q-table cell is the expected maximum future reward for a state and an action. The Q-table provides the desired values (often 0) before the agent searches the environment. The Q-function receives state and action as inputs. It then returns the expected future reward for that state and action according to the Eq. (1). By searching the agent in the environment, the Q-table provides a better estimate by recursively updating Q (s, a). The general process of the Q algorithm shows in Fig. 4.

Each step in the diagram of Q-learning is as follows:

Step 1: Initialize the Q-table: Initialize Q-table with zero value. There are n = 1600 rows, where n is the number of states. There are m = 4 columns, where m is the number of actions.
Step 2: Start an episode and select a state to start: An episode is a process in which the agent starts from one state and continues until it reaches the endpoint. The number of episodes is 20,000. The final point is the location of the gamma source.
Step 3: Choose an action: Select an action "a" in the current state of "s," which is estimated based on the present value of the Q-value. However, since all Q-values are zero initially, the "epsilon greedy strategy" must be used. An "epsilon" search rate is determined, which is initially set to one. These are the rates of steps that the agent takes at random. In the beginning, this value should be equal to the maximum possible value because the agent knows nothing about the Q-table values. A random number is generated if it is greater than the epsilon, extraction is performed (this means that the best action is selected by the present knowledge or Q-table). Otherwise, the search will take place (this means that the best action is selected randomly)^33^. After the agent's estimating Q-values increases, gradually, the epsilon value decreases. The epsilon search rate was initially set to 1 and then decreased by a factor of 0.01.
Step 4: Take action a, observe reward and next state: View the reward based on where the agent was and what they did. Reward +0.5 will be received if the agent approaches the gamma source, and if the agent moves farther, it will be received a − 1.5 penalty.
Step 5: Update Q(s,a) value: Update the value of Q (s, a) for state s and operation a.
Step 6: Observe the next state: Go to the next state. If the next state is the gamma source location, the agent will be received a reward of 100. Otherwise, repeat steps 3 to 6 until the agent finds the gamma source location and ends the episode.
Step 7: Start new episode: Increase the number of episodes by one.
Step 7: End of learning: If the number of episodes is not equal to 20000, go to step 1; otherwise, end the learning and print Q-table.

In each episode, the agent passes from different states to reach the goal and gets a reward or penalty in each state. The total reward is the sum of rewards that the agent earns in each episode^33^.

Note that the one hundred rewards are received when the agent finds the lost gamma source, and the amount of negative reward is three times the positive reward; therefore, the correct actions (move closer to the source) are much more than the wrong actions.

When the agent does not know the environment, moves randomly in the initial episodes and does the wrong action. Therefore, the total reward becomes negative because of getting more penalties. In the following episodes, with previous experience and better learning of environment, the total reward becomes positive because of getting more rewards.

## Proposed algorithm using CNN and Q-learning methods

Table 1, shows that the CNN accuracy is low for locating lost gamma sources in the room with a maze. To increase the accuracy, we propose to use the Q-learning algorithm along with the CNN method. Two facts are used to increase the accuracy of finding the lost gamma source, 1: the longer the path length, the greater the accuracy, and 2: the closer the trail to the location of the gamma source, the more accurate it is. Q-learning helps the agent get close to the lost gamma source and path length increase optimally; therefore, the accuracy increases. So that navigation can be performed while the accuracy of locating the gamma source increases.

**Figure 5:**
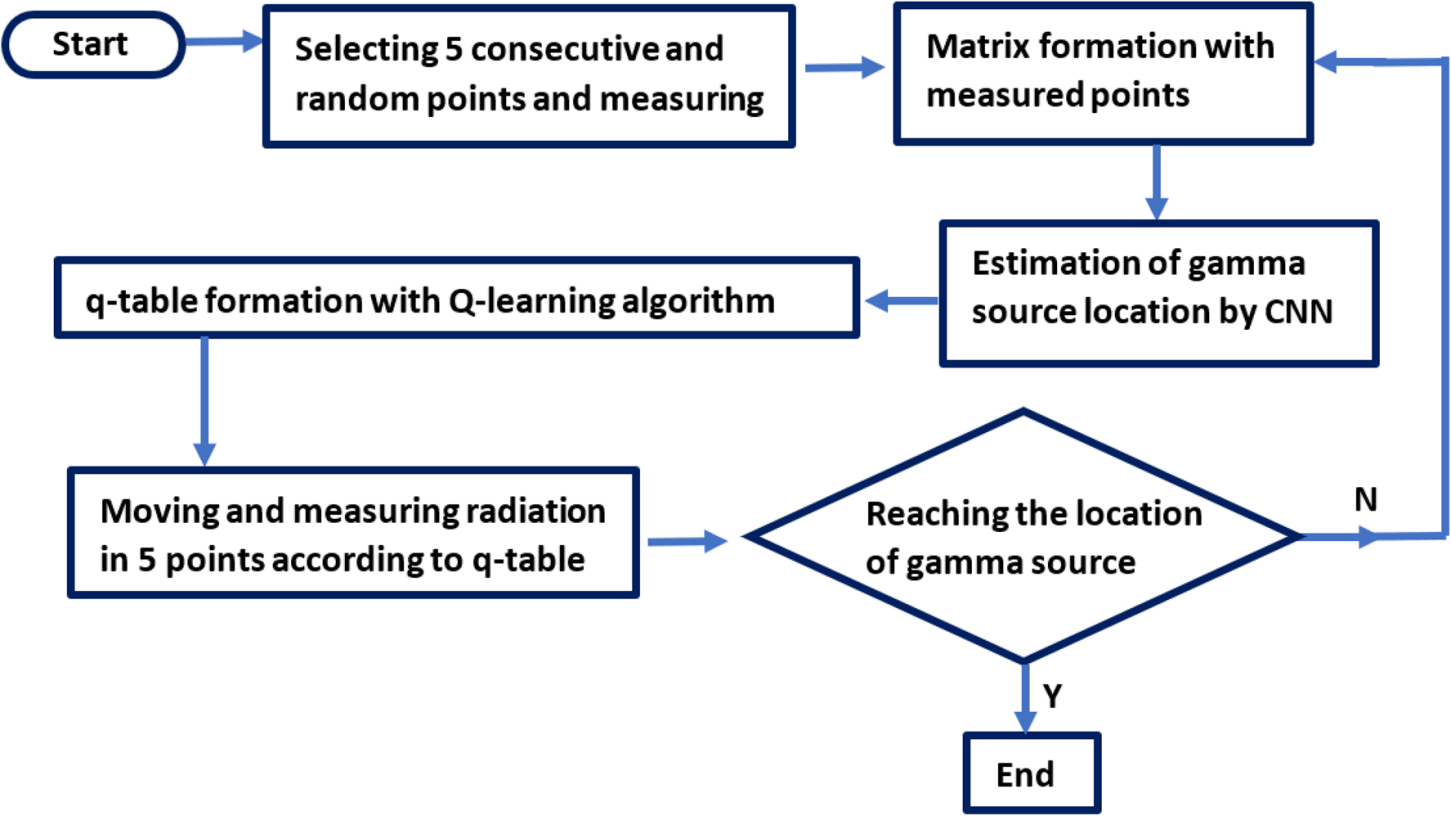
Diagram of the proposed algorithm using Q-learning and CNN.

**Figure 6:**
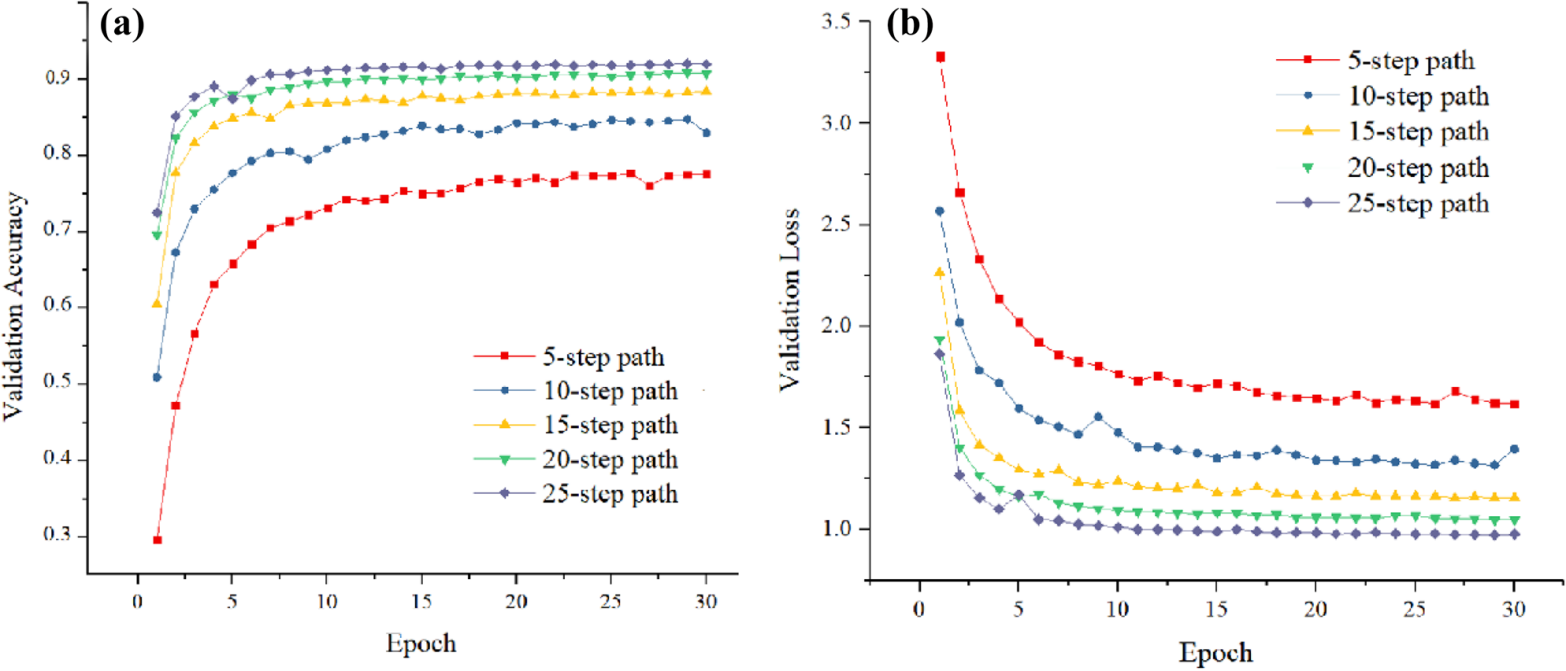
(**a**) Validation Accuracy and (**b**) Validation Loss versus Epoch for different step paths.

The procedure is shown in the form of a diagram in Fig. 5. The starting point of the search process is fixed at the entrance of the room. The agent starts from this point and moves along the wall to receive non-zero radiation. As soon as non-zero data is recorded, the energy deposition was measured at five random points along a path of 200 cm. After measuring energy deposition in a 5-step-path, a radiation measurement matrix is constructed (40 × 40 matrix with five non-zero elements at energy deposition measurement points. The rest of the elements are zero, and the elements correspond to the location of the walls are one). The radiation measurement matrix is the CNN input. The CNN estimates the location of the gamma source. The estimated location of the gamma source is given to the Q-learning algorithm. According to the Q-learning table, five new measurements are measured to get closer to the gamma source. After this step, the agent records ten measurements as a 10-step-path matrix, which will give to CNN again as input. The CNN re-estimates the gamma source location with a higher percentage of accuracy. This process continues until a 25-step path is formed, unless the agent reaches the location of the gamma source in one of the previous steps. Paths with 5, 10, 15, 20 and 25 step lengths are made using the mentioned method, and the CNN is trained with a new dataset for a room with a walled maze with a 200 cm thickness. Figure 6, shows the accuracy and loss functions for the train data of different length paths.

The validation data is the samples that were not shown to the network during training. The validation accuracy refers to how much the CNN works for cases outside the training set. The closer the accuracy trend to one, the better the network performance.

During an epoch, the loss function is calculated, and the loss graph shows whether the network parameters are well selected. The trend should be toward zero. The loss curve during training is used to debag a CNN. It is a snapshot of the training process, and more insight can be obtained. The graph of loss versus epoch should be a downward trend, and the closer it is to zero, the better. According to this description, Fig. 6 shows that CNN is well trained.

The results of the CNN evaluation with test data are given in Table 2, for a room with a 200 cm walled maze.

**Table 2:** The evaluation results of CNN model wall mazed thickness L_1_ = L_2_ = 200 cm.

Step	Precision	Recall	F1-score	Accuracy
5	0.73	0.71	0.72	0.71
10	0.82	0.80	0.81	0.80
15	0.85	0.84	0.85	0.84
20	0.88	0.86	0.87	0.86
25	0.93	0.92	0.92	0.92

The CNN with 25-step-path can detect the location of the gamma source with a high accuracy of 90% and move according to the Q-learning table until it reaches the gamma source. Without using the Q-learning, the gamma source location can be predicted with 65% accuracy with a 25-step-path, while using the Q-learning, the accuracy of %71 is achieved just with 5-step-path. Therefore, finding the lost gamma source with the same accuracy requires a longer path and more measurement points, and so more time, if just CNN is used. At each stage, while forming a longer path, the agent gets closer to the gamma source, which helps the measurement process be carried out optimally to route and increase accuracy. A comparison of Tables 1 and 2, shows that using the Q-learning increases the accuracy more than 20%.

**Figure 7:**
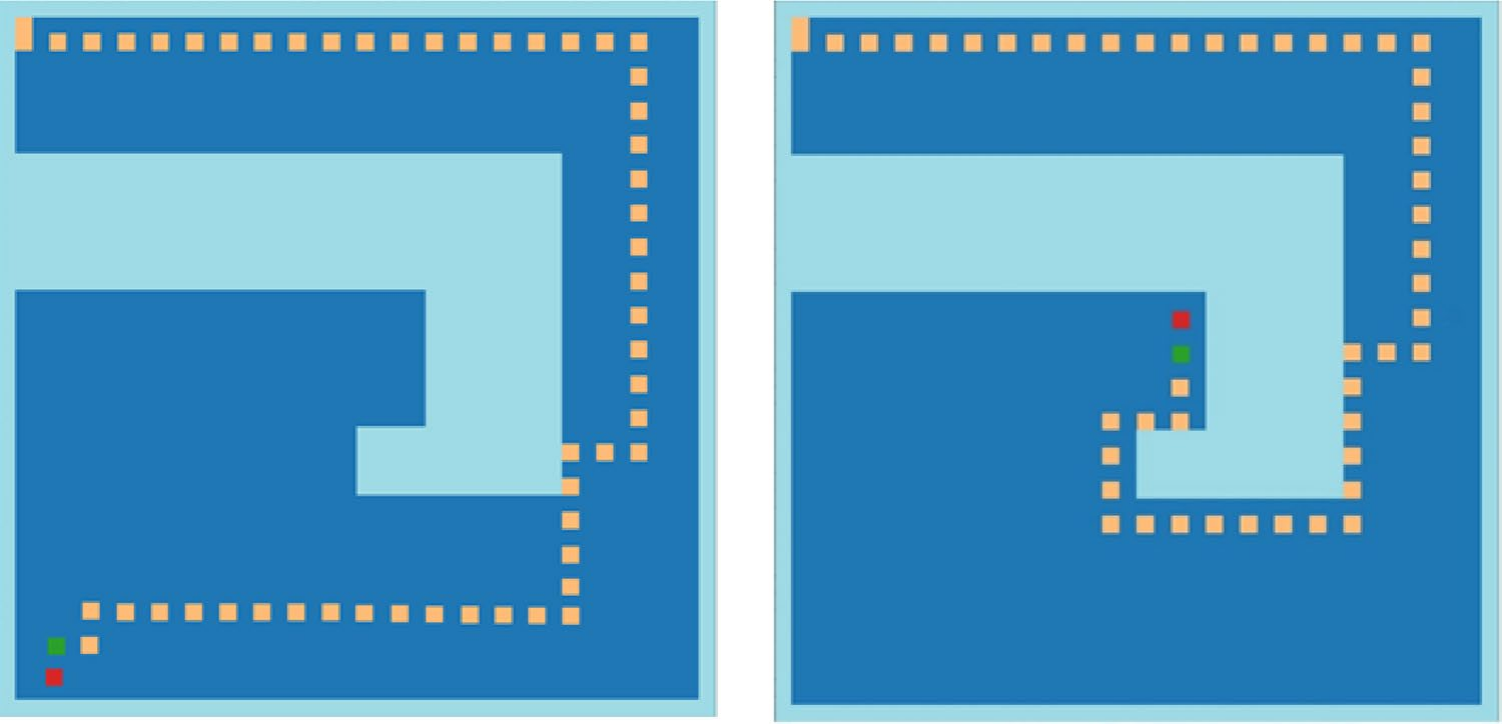
Examples of routing performed by the proposed algorithm.

Examples of routing performed by the proposed algorithm are given in Fig. 7. The agent starts moving from the room entrance (bottom left of the room in the figures) to reach the gamma source (red cell in the figures).

In both examples, the source and the agent are at two different sides of the wall at the beginning of the exploration. The wall thickness is such that the agent does not receive any radiation at the beginning of the exploration. The proposed method helps the agent recognize that the gamma source is behind the wall and moves toward it. Also, the examples show that the agent finds the source with the slightest error in routing. The Q-learning algorithm detects the environment and helps the CNN purposefully record the deposited gamma radiation energy in different places and use them to predict the gamma source location with appropriate accuracy.

## Conclusions

Finding a lost gamma source is facing challenges in a room with a thick maze. The wall thickness of the maze in nuclear facilities is such that it dramatically attenuates or blocks gamma radiation. Therefore, the scattering and distribution of radiation do not have a specific pattern. In this type of room, measuring radiation and following the radiation distribution pattern is not accurate enough to find lost gamma sources. Therefore, using a method in which the agent can identify the environment geometry and then search the gamma radiation to simultaneously estimate the location of the gamma source and routing toward the gamma source can be a suitable method in this room.

In this paper, a room with a walled maze with different thicknesses (50 cm, 100 cm and 200 cm) has been simulated containing a lost gamma source in the Geant4 simulation program. The maps of gamma radiation energy deposition have been simulated for different gamma source locations. Different datasets for CNN training have been obtained by constructing different trails of sequential measurements of radiation energy deposition from radiation energy deposition maps. The CNN has trained for 5, 10, 15, 20 and 25 step paths. The results show that the CNN estimates the lost gamma source location by 48 percent accuracy with a 5-step path and 65 percent accuracy with a 25-step path for 200 cm walled maze. Therefore, the CNN alone could not be a precise algorithm for finding lost gamma sources in this geometry. This paper proposes using CNN and Q-learning simultaneously to locate and route a lost gamma source in the room with a thick-walled maze. The measurements of gamma radiation energy deposition were done optimal such that the trail of measurement became closer to the gamma source location by using Q-learning. The Q-learning algorithm identifies the environment by performing different episodes and determines the appropriate path for getting closer to the gamma source from any point to the estimated location of the gamma source.

Based on the proposed method, after every five measurements of radiation energy deposition, the location of the gamma source is estimated by CNN. Then the agent moves five steps towards the estimated gamma source location by the Q-learning algorithm and measures energy deposition in each step. Again, CNN estimates gamma source location by new and old measurements of radiation energy deposition. The agent gets closer to the lost gamma source while the probability of estimation of its location increases. In all steps, the distance between the sequential points of radiation measurements is considered 50 cm. The CNN accuracy of estimating the lost gamma source location increases from 48 to 71% for 5-step path and from 65 to 92% for 25-step path for walled maze with 200 cm thickness. The proposed method estimates the Gamma source location with a 5-step path more accurately than only the CNN method with a 25-step path, which saves time. Since the agent gets closer to the lost gamma source while measuring the gamma energy deposition in a path, locating the lost gamma source and routing is done simultaneously with high accuracy.
